# Policy for rare diseases

**DOI:** 10.1038/s43856-023-00254-4

**Published:** 2023-02-28

## Abstract

Professor Bobby Gaspar is a distinguished physician-scientist who is a thought leader in translating basic research from bench-to-bedside and strategic work that facilitated bringing life-saving therapies to patients with rare diseases. He has over 30 years of experience in pediatric medicine working in the NHS and the biotechnology sector, and is the founding member of Orchard Therapeutics, where he serves as Chief Executive Officer. In this Q&A, Professor Gaspar provides insight into the regulatory approval and policy considerations for bringing novel therapies for rare diseases from discovery through to clinical application.

**Figure:**
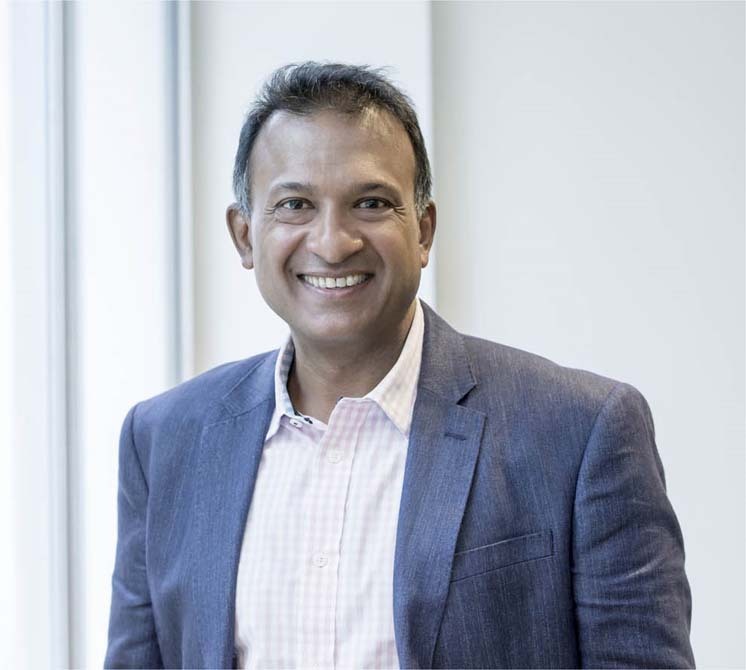
Photo courtesy of Orchard Therapeutics. All rights reserved.

Please tell us a bit about yourself and your interest in this area?

I have been involved with hematopoietic stem cells (HSC) gene therapy for rare diseases as a physician-scientist for nearly three decades and worked on some of the earliest research and clinical trials in this field. I am incredibly proud of how far this therapeutic approach has come and the promise it holds for many devastating, difficult-to-treat diseases.

However, it quickly became apparent to me that to broaden access to this pioneering science, we needed to partner with other entities that could help translate our early clinical successes to medicines that could be approved by regulatory authorities and delivered through a commercial infrastructure to patients in need.

After my many years in academia and treating children living with rare and severe immune deficiencies, I co-founded Orchard Therapeutics in 2015 with the goal of unlocking the full potential of this therapeutic modality. Within just a few years, we had established a balanced portfolio of investigational HSC gene therapies at different stages of development through various academic partnerships and our own in-house discovery capabilities.

Through Orchard, I also believe, we have the ability to fundamentally change the landscape of some devastating diseases by not only developing potentially curative therapies but also by facilitating the implementation of newborn screening for these conditions. By diagnosing at birth and then treating with a potentially transformative therapy, we can essentially change the course these conditions and save children, their families and the healthcare system of the devastating consequences.

How does your position as CEO of Orchard Therapeutic alongside your NHS role enable you to contribute to the development of new policy for the rare disease community?

Having been a physician seeing patients, researcher, and Chief Executive Officer of a biopharmaceutical company, I have seen first-hand the different aspects required to deliver potentially transformative medicines to patients living with rare diseases.

In that time, I’ve come to the realization that changing the course of disease takes much more than scientific breakthroughs — although they are foundational. I know how all-consuming and fraught research and discovery can be, but I also know that the remaining steps of regulatory approval, manufacturing and reimbursement are equally complex.

It is only with a holistic view of the drug development ecosystem and care continuum that we can make policy advances that truly broaden access to meaningful medical accomplishments on behalf of people living with rare diseases, while also ensuring the long-term sustainability of healthcare systems.

As pioneers in this new and developing area, we can influence practice and policy. This stretches from how we design clinical trials, interact with regulators, build the necessary commercial infrastructure, and influence the reimbursement landscape to enable access to patients. The ability to partner with stakeholders in both the therapeutic area and in developing newborn screening allows us to shape policy and practice for how these diseases will be treated and accessed in the future.

What are the existing roadblocks in effective development and implementation of new policy for patients with rare diseases?

Particularly in rare diseases, it takes many different stakeholders to bring a potential therapy forward for patients. From the clinicians performing basic research to understand the natural history, epidemiology, and genetic drivers of the disease; to the academic medical institutions translating those insights into scientific breakthroughs; the advocacy organizations raising awareness, galvanizing the community, and coordinating research efforts; the companies carrying the mantle of translating a program from academic- to commercial-stage processes necessary to secure regulatory approval and build the infrastructure to deliver the therapy to patients; as well as the financial backers who make much of that critical work possible.

While each stakeholder group has a unique role and competency critical to advancing the process, their interests are not always completely aligned or balanced across the discovery and development process. Moreover, each have their own pain points and challenges they need to address.

Looking at the landscape, there are some extremely important areas that need to be addressed if we are to bring forward these therapies in a timely manner. Foremost, we need to speed up drug development timelines as patients cannot wait and suffer, especially when there are no alternative treatment options, and a therapy has shown a favorable risk/benefit profile in a clinical setting. This means working with regulators to find the best pathway to bring these therapies towards approval. We need to find the optimal study designs and the appropriate control arms, given that in some rare diseases there are no approved standard therapies available. We also have to work with reimbursement agencies to articulate the value of these medicines and make them accessible, while ensuring there is value for patients and healthcare systems. Finally, for rare diseases, early diagnosis is paramount—especially in conditions where intervention before disease onset is crucial, such as metachromatic leukodystrophy, which causes rapidly progressing, irreversible, and in the most severe forms, ultimately fatal neurodegeneration in children. Many of these conditions are currently not screened for because there is no treatment available. But with the advent of new therapies, screening is essential and for this reason, we must overcome the hurdles and delays associated with implementing newborn screening and engage with national screening agencies.

The disparate nature of the ecosystem is one of the barriers to the effective development and implementation of effective new policies for rare diseases. Which is why we must commit to keep working together to create a sustainable path forward for more innovations and to truly make therapeutic advances accessible and viable for as many patients as possible.

What type of research-based evidence is needed to make pertinent policy for the rare disease community?

Ultimately, we need to apply learnings earlier in the development process so we can better anticipate potential upstream barriers. For example, as the science evolves, so do regulatory requirements, manufacturing and controls, as well as reimbursement. Enabling researchers to apply insights earlier in the process means we can better translate groundbreaking science and target the right resources needed to get a therapy approved and make it available to the populations who may benefit from it the most.

This means we need to assess the treatment landscape consistently and use these lessons to drive development strategies. For example, the standard of care is always evolving, which can influence prescriber and patient preferences, and this in turn affects value assessment and funder decisions. This evolving landscape must be consistently evaluated to guide stakeholder decisions around research, funding, and commercialization. It’s also the case that for some diseases where no approved therapy exists, we may need to use the normal course of the disease as a comparator. This evidence needs to be collected in a manner that is acceptable and has buy-in from regulatory agencies.

Furthermore, if we want more rare disease treatments to make it to the market, regulatory agencies will need to be more open to flexibility and partnership. Regulatory science has definitively improved, but a tremendous amount of work is required to move from early academic research and discovery to at-scale commercial manufacture—including complex and resource-intensive comparability studies, mock shipments, manufacturing process optimization, and more. When that work must be repeated across different regulatory regimes globally, the need for regulatory harmonization becomes self-evident.

Finally, the use of newborn screening pilot studies in different geographies can provide the collective evidence on feasibility, false positive rate and diagnostic accuracy that can be taken to agencies for national implementation.

How can researchers, clinicians, policy makers and funders work together to develop worthwhile policy for rare diseases?

No single stakeholder can solve all the challenges facing the rare disease community. Instead, all stakeholders will need to undertake honest, critical conversations to apply the downstream learnings and experiences we have gained throughout the development and commercialization process, as these insights have an impact on the resources needed to get a therapy approved and to patients in need.

These stakeholders need to have discussions about the impact of these challenges on our ability to bring gene therapy treatments forward, including when and how.

How can we ensure policies are optimized to serve the needs of patients with rare diseases in the future?

This is of critical importance given the speed of scientific innovation continues to outpace the policy changes necessary to create a sustainable path forward for advanced medicines.

Nowhere is this more apparent than the field of cell and gene therapy—which holds tremendous potential to essentially cure many different monogenic rare diseases. Widely recognized as one of the most pioneering treatment modalities, we now sit at a critical inflection point between promise and widespread practice.

Despite recent clinical, regulatory and early commercial breakthroughs and successes, we now see potentially class-limiting warning signs, with seven of the twenty four approved treatments having been pulled from the market in Europe according to the Alliance for Regenerative Medicine, and mostly for reasons other than efficacy or safety.

To help mitigate this, we need to ensure that even early in the development process, a thorough assessment of the development, manufacturing, commercial pathway and potential has been made to avoid disappointment further down the line. The potential of these curative therapies is enormous but that promise can only be realized if there is a path forward not only for approval but also for reimbursement, diagnosis and treatment of sufficiently large numbers of patients for commercial sustainability.

Ensuring these breakthroughs ultimately reach patients in need requires systemic changes in how these medicines are delivered and reimbursed—moving from chronic treatment models to also enabling the administration of one-time potentially curative therapies.

